# Absinthe Drinking in Paris

**Published:** 1868-09

**Authors:** 


### ARTICLE XI.

### *Absinthe Drinking in Paris.*

The indulgence in absinthe, which already prevails to a great extent among all classes of Frenchmen, threatens to become as widespread in France and as injurious there as opium-eating is in China. If a visitor to Paris strolls along the boulevards from the Madeline to the Bastile some summer's afternoon between five and six o'clock—which is commonly called the "hour of absinthe"—he can hardly fail to remark hundreds of Parisians seated outside the various cafes or lounging at the counters of the wine shops and imbibing this insidious stimulant. At particular cafes, the Cafe de Bade, for example, out of fifty idlers seated at the little round tables forty-five will be found thus engaged. But it is not on the boulevards alone that absinthe is the special five o'clock beverage. In most of the wine-shops in the faubourgs, in the Quartier Latin, and round about the Ecole Militaire, you may see at that particular hour workmen, students, soldiers, clerks, charbonniers, chiffonniers even, mixing their customary draughts of emerald-tinted poison, and watching the fantastic movements of the fluid as it sinks to the bottom of the glass, wherein it turns from green to an almost milky white, at the moment when the perfumes of the various aromatic plants from which it is distilled disengage themselves. A quarter of a century ago absinthe was the drink of French coachmen, grooms, and footmen, and people of the lowest class; to-day its most ardent lovers are to be found among educated and well-to-do Parisians. Literary men, professors, artists, actors, musicians, financiers, speculators, shopkeepers, even women yield themselves up to its seductive influence, to those indefinable provocations which seem they say, to impart renewed activity to an enfeebled brain, developing a world of new ideas, and which thus, it is believed, have inspired many a noble work of imagination in literature and art. It may be so, but then those who habitually excite the brain with absinthe soon discover that they can produce positively nothing without its aid, and that a time arrives when heavy stupor supersedes that excitement of the intellectual faculties which once seemed so easy and so harmless. After the first draught of this poison, which Dr. Legrand, who has studied its effects, pronounces it to be one of the greatest scourges of our time, you seem to lose your feet, and you mount to a boundless realm without a horizon. You probably imagine that you are going in the direction of the infinite, whereas you are simply drifting into the incoherent. Absinthe affects the brain unlike any other stimulant; it produces neither the heavy drunkenness of beer, the furious inebriation of brandy, nor the exhilarant intoxication of wine. It is an ignoble poison, destroying life not until it has more or less brutal-ized its votaries, and made drivelling idiots of them. There are two classes of absinthe drinkers. The one, after becoming accustomed to it for a short time, takes to imbibing it in considerable quantities, when all of a sudden delirium declares itself. The other is more regular, and at the same time more moderate in its libations; but upon them the ef fects, though necessarily more gradual, are none the less sure. Absinthe drinkers of the former class are usualy noisy and aggressive during the period of intoxication, which, moreover lasts much longer than drunkenness produced by spirits or wine, and is followed by extreme depression and a sensation of fatigue which are not to be got rid of. After awhile the digestive organs become deranged, the appetite continues to diminish until it is altogether lost, and an intense thirst supplies its place. Now ensues a constant feeling of uneasiness, a painful anxiety, accompanied by sensations of giddiness and tinglings in the ears; and as the day declines, hallucinations of sight and hearing begin. A desire of seclusion from friends and acquaintances takes possession of the sufferer, in whose countenance strong marks of disquiet may be seen : his mind is oppressed by a sort of melancholy, and his brain affected by a sluggishness which indicates approaching idiocy. During its more active moments he is continually seeing either some imaginaay persecutor from whom he is anxious to escape, or the fancied denunciator of some crime he dreams he has committed. From these phantoms he flies to hide himself, or advances passionately towards them protesting his innocence. At this stage the result is certain, and dissolution is rarely delayed very long. The symptom that first causes disquiet to the habitual absinthe drinker is a peculiar affection of the muscles, commencing with fitful contractions of the lips and muscles of the face, and trembling in the arms, hands, and legs. These are presently accompanied by tinglings, numbness, and a distinct loss of physical power; the hair falls off, the countenance becomes wan and sad looking, the body thin, the skin wrinkled and of a yellowish tinge—everything in short indicates marked decline. Simultaneously with all this, lesion of the brain takes place; sleep becomes more and more disturbed by dreams, nightmares, and sudden wakings; ordinary illusions, succeeded by giddiness and headaches, eventually give place to painful hallucinations, to delirium in its most depressing form, hypochondria, and marked impediment of speech. In the end come entire loss of intellect, general paralysis, and death. There are two kinds of absinthe consumed in Paris, the common and the the Swiss absinthe, the latter of which possesses almost double the intoxicating properties of the former. A few years ago the consumption of the common absinthe was three times that of the Swiss ; but now the proportions are reversed, and four times as much Swiss absinthe is drunk as of the common quality. According to official statistics, France receives from Switzerland nearly 2,000,000 gallons of the noxious compound annually : in addition to which an enormeous quantity made in Paris is sold as the veritable Swiss production. Genuine absinthe is distilled from the leaves of major and minor absinthe, angelica roots, calamus, aromaticus, aniseed, dittany leaves, and wild marjoram, all of which have been previously bruised and soaked for a period of eight days in alcohol. A quantity of oil of aniseed is then added, and the whole is carefully mixed together. Occasionally fennel, mint, etc., enter into the composition. The utmost care is taken to obtain the right shade of color, and to insure the liquid expanding and whitening well when mixed with water. Should it prove to be deficient in these qualities, the manufacturer does not hesitate to add indigo, hyssop, nettles, and even to have recourse to sulphate of copper, to obtain the precise tint of green, or chloride of antimony, to produce the muddy-white precipitate, both of these chemicals being deadly poisons. Paris actually has its clubs of absinthe drinkers, the members of which are pledged to intoxicate themselves with no other stimulant, and even to drink no other flnid—the only pledges, it is believed, which they do not violate. They assemble daily at some appointed place of rendezvous at a certain hour, and proceed to dissipate their energies and their centimes in draughts of that fatal poison which fills the public and private madhouses of Paris. These absinthe-drinking clubs are certainly not numerous, but liquor-shops abound in all quarters of the city where absinthe may be said to be the staple drink, and lately several have sprung up which, to attract the youth of Paris to them, dispense the insidious beverage at the hands of pretty women. In the French army drinking of absinthe of the cheapest quality, and as a matter of course the most deleterious of all, used to prevail to such an extent that both military and medical commissions were appointed to report upon the practice and the effects resulting from it. The facts were so alarming that the goverment not only formally interdicted its consumption, but made every endeavor to keep it beyond the reach of the soldiers. In Paris and other garrison towns these efforts were not particularly successful; but it fared hard with any camp followers of expeditionary corps in Algeria, or at Chalons, or other parts of France where temporary camps were formed, who chanced to be detected in supplying absinthe to the troops. In the French navy its consumption is rigidly prohibited, not merely to the common seamen but to the officers as well.—*Pall Mall Gazette.*

### MONTHLY SUMMARY.

